# Predicting acute kidney injury with an artificial intelligence-driven model in a pediatric cardiac intensive care unit

**DOI:** 10.1186/s44158-023-00125-3

**Published:** 2023-10-18

**Authors:** Tiziana Fragasso, Valeria Raggi, Davide Passaro, Luca Tardella, Giovanna Jona Lasinio, Zaccaria Ricci

**Affiliations:** 1 Pediatric Cardiac Intensive Care Unit, Bambino Gesù Children’s Hospital, IRCCS, Piazza S.Onofrio 4, 00165 Rome, Italy; 2 Department of Statistical Sciences, Sapienza - University of Rome, Rome, Italy; 3 Pediatric Intensive Care Unit, Department of Anesthesia and Critical Care, Meyer Children’s University Hospital, IRCCS, Florence, Italy; 4 Department of Health Sciences, Section of Anesthesiology and Intensive Care, University of Florence, Florence, Italy

**Keywords:** Acute kidney injury, Machine learning, Pediatric cardiac intensive care unit, Risk factors, Predictive models, Electronic health record

## Abstract

**Background:**

Acute kidney injury (AKI) is among the most common complications following cardiac surgery in adult and pediatric patients, significantly affecting morbidity and mortality. Artificial Intelligence (AI) with Machine Learning (ML) can be used to predict outcomes. AKI diagnosis anticipation may be an ideal target of these methods. The scope of the study is building a Machine Learning (ML) train model with Random Forest (RF) algorithm, based on electronic health record (EHR) data, able to forecast AKI continuously after 48 h in post-cardiac surgery children, and to test its performance.

Four hundred nineteen consecutive patients out of 1115 hospital admissions were enrolled in a single-center retrospective study. Patients were younger than 18 years and admitted from August 2018 to February 2020 in a pediatric cardiac intensive care unit (PCICU) undergoing cardiac surgery, invasive procedure (hemodynamic studies), and medical conditions with complete EHR records and discharged after 48 h or more.

**Results:**

Thirty-six variables were selected to build the algorithm according to commonly described cardiac surgery-associated AKI clinical predictors. We evaluated different models for different outcomes: binary AKI (no AKI vs. AKI), severe AKI (no-mild vs severe AKI), and multiclass classification (maximum AKI and the most frequent level of AKI, mode AKI). The algorithm performance was assessed with the area under the curve receiver operating characteristics (AUC ROC) for binary classification, with accuracy and* K* for multiclass classification. AUC ROC for binary AKI was 0.93 (95% CI 0.92–0.94), and for severe AKI was 0.99 (95% CI 0.98–1). Mode AKI accuracy was 0.95, and *K* was 0.80 (95% CI 0.94–0.96); maximum AKI accuracy was 0.92, and *K* was 0.71 (95% CI 0.91–0.93). The importance matrix plot demonstrated creatinine, basal creatinine, platelets count, adrenaline support, and lactate dehydrogenase for binary AKI with the addition of cardiopulmonary bypass duration for severe AKI as the most relevant variables of the model.

**Conclusions:**

We validated a ML model to detect AKI occurring after 48 h in a retrospective observational study that could help clinicians in individuating patients at risk of AKI, in which a preventive strategy can be determinant to improve the occurrence of renal dysfunction.

**Supplementary Information:**

The online version contains supplementary material available at 10.1186/s44158-023-00125-3.

## Background

Acute kidney injury (AKI) is one of the most common complications following cardiac surgery in adult and pediatric patients and affects short- and long-term morbidity and mortality [1, 2].

The incidence of AKI in post–cardiac surgery children ranges from 15 to 80%, depending on the center, AKI definition used, and the population studied [3]. The incidence of AKI in our center is about 70%, of which 26% of cases are classified as mild and 43% as severe AKI [4].

All cardiac surgical patients are exposed to inflammatory (e.g., cardiopulmonary bypass, CPB) and ischemic triggers (e.g., aortic cross-clamp) along with nephrotoxins (e.g., antibiotics and contrast media), which hamper proper renal perfusion [5]. Furthermore, children have peculiar conditions predisposing them to AKI, e.g., pulmonary hypertension and cyanotic heart disease, which occur in non-surgical patients and throughout all pediatric cardiac intensive care unit (PCICU) admissions [2]. All these factors can be present in the postoperative phase, but AKI can also occur in non-surgical patients and throughout all PCICU admissions. Late presentation of severe AKI may have a significantly higher mortality and be strictly related to baseline cardiac disease [4].

Prevention has been identified in the adult setting as an effective approach to reduce AKI incidence. The introduction of the Kidney Disease Improving Global Outcome (KDIGO) AKI bundle, applied to prevent post-cardiac surgery AKI, included utilization of biomarkers, optimization of volume status and hemodynamics, avoidance of nephrotoxic drugs, and prevention of hyperglycemia [6] and has shown promising results in adult patients. Similarly, interventional trials exist for the treatment of established post-cardiosurgical AKI; a meta-analysis based on available observational data did not identify any effective candidate interventions [7]. Therefore, in the pediatric population, every effort must be posed to prevent AKI.

In the last 10 years, artificial intelligence (AI) has become a hot topic in medical research. The concept of AI refers to the development of computer systems capable of performing tasks normally requiring complex calculations and multiple regressions. The ability of AI appears particularly suitable when the prediction of a very well-defined outcome is concerned [8]. Machine Learning (ML) is a field of applied AI that allows software applications to run very specific types of data. The predictions that ML algorithms make are based on the recognition of recurrent patterns of data.

The aim of this research is to develop an algorithm (Random Forest, RF) able to predict AKI episodes defined according to the KDIGO stage, after 48 h, throughout all PCICU admissions.

Although the efficacy of prediction models has been already investigated in several studies, we conducted the present study to evaluate the performance of a machine learning model in a population with well-known and homogeneous risk factors and to see any fluctuation performances in predicting different severity of AKI.

## Methods

### Study design and settings

The study was conducted following the Strengthening the Reporting of Observational Studies in Epidemiology (STROBE) statement [9].

The data used in this study were retrospectively extracted from the electronic health record (EHR) system running in the PCICU at Bambino Gesù Children’s Hospital between January 1, 2018, and February 29, 2020. All data were extracted and processed in a de-identified format.

Bambino Gesù Ethics Committee approved the study design and waived the need for informed consent due to the retrospective nature of the study (protocol number 2002_OPBG_2019).

### Inclusion and exclusion criteria

Patients from birth to 17 years old who required elective and urgent admission to the PCICU for surgical, invasive procedures (hemodynamic studies), and medical conditions were enrolled. Therefore, all patients older than 18 years at admission were excluded. All admissions lasting less than 48 h were excluded. Patients without invasive arterial pressure monitoring were also excluded since non-invasive arterial pressure monitoring may show significant differences with invasive measurements [10]. Also, patients with a clinical history of chronic renal dysfunction were excluded.

### AKI definition

AKI was defined according to the KDIGO classification [11]. KDIGO uses gradual increases of serum creatinine (SCr) and progressive decrements in urine output (UOP) to define AKI and its severity, staging AKI from 0 (no AKI) to 3. Stages 2 and 3 are considered as severe AKI, whereas 1 is defined as mild.

In our PCICU, an automatic KDIGO calculation has been implemented in the EHR to generate a KDIGO stage at the time of admission, eventually updating every hour. Detected AKI episodes were extracted for the purpose of this study. Basal creatinine, to which the increase in SCr is referred, was considered in all patients as the pre-PCICU admission value, that was available in all patients.

### Data collection

The selection of variables to build the predictive model was guided by the availability of parameters validated and stored in the EHR and through previously described predictors of AKI in cardiosurgical patients (Table 1) [12].

**Table 1:** Variables collected for algorithm building

Type of variable	Name of variable
Admission and post-admission data (fixed data)	Sex, age, weight, PIM3, VIS, admission type, basal SCr, CPB duration, cross-clamp duration
Vital signs	SAP, DAP, MAP, O_2_ saturation, HR
Fluids	Diuresis, fluid input, fluid output, blood input, blood output
Blood gas analysis	BE, Na + , Cl-, Lactate, blood pH
Laboratory analysis	SCr, albumin, hemoglobin, platelets, LDH, aPTTs
Therapies	Adrenaline, milrinone, furosemide, levosimendan, vasopressin, ethacrynic acid

*Admission and post-admission data* and *Fluids* are manually inserted into the electronic health record (EHR) by clinicians and nurses. *Vital signs*, *Blood gas analysis*, and *Laboratory analysis* are automatically collected from monitors and labs into the EHR. *Therapies* are prescribed by clinicians and validated by nurses. Once they are validated, data are automatically extracted from the EHR

*PIM3* Paediatric Index of Mortality3 score, *VIS* Vasoactive Inotropic Score, *Admission type* post-surgical/post cath lab/medical admission, *CPB* cardiopulmonary bypass, *SCr* serum creatinine, *SAP* systolic arterial pressure, *DAP* diastolic arterial pressure, *MAP* mean arterial pressure, *HR* heart rate, *BE* base excess, *LDH* lactic dehydrogenase, *aPTTs* activated partial thromboplastin time in seconds

Datasets used to train and validate the algorithm were obtained from Digistat® EHR (Ascom UMS srl Unipersonale, Scandicci, Florence, Italy) utilized in our center; it applies Structured Query Language (SQL). Medical admissions’ data were extracted between PCICU admission and discharge; surgical and cath lab admissions were extracted between the end of the first surgery (or first cardiac procedure) and PCICU discharge or the start of the following procedure (in case of repeated interventions). In cases where cath lab admissions were followed by surgery, data were included between PCICU admission after the cath lab procedure and discharge after surgery. Data on Digistat were both manually inputted and automatically transmitted from electronic devices and monitors. All data were validated by nurses and doctors or provided by the laboratory.

Demographic data at admission, baseline vital signs, and laboratory values were obtained. After PCICU admission, vital parameters were gathered for analysis every 2 h (as per nurses’ validation according to institutional policy), and laboratory exams were requested every 24 h. The first blood gas analysis (BGA) per each PCICU day and preselected therapies likely associated with AKI risk were recorded for the analysis. Table 1 details demographic, clinical, laboratory, BGA, and therapeutic variables that were collected for this study. Also, PCICU length of stay and patients’ mortality are reported in Table 2.

**Table 2:** Demographics and outcomes

Variables		ALL (*N* = 419)		No/mild AKI (*N* = 196)		Severe AKI (*N* = 223)		*p*
		**Median/[n.]**	**(IQR)**	**Median/[n.]**	**(IQR)**	**Median/[n.]**	**(IQR)**	
**Age**	**Days**	164	(31–999)	194	(39–699.25)	157	(22–1386.5)	0.92
**Neonates**	**[n.]**	[105]		[45]		60]		0.91
**Weight**	**Kg**	5.6	(3.3–12.5)	5.6	(3.4375–10.075)	5.5	(3.2–14)	0.81
**Gender**	**F [n.]**	[193]		[91]		102]		0.93
	**M [n.]**	[226]		[105		121]		
**PIM3**		0.0153	(0.009–0.039)	0.013	(0.009–0.035)	0.020	(0.010–0.045)	0.003
**VIS**		10	(0–15)	7.5	(0–15)	10	(2.5–15)	0.015
**basal SCr**	**(mg/dl)**	0.35	(0.26–0.58)	0.31	(0.26–0.5)	0.395	(0.26–0.6225)	0.017
**CBP duration**	**Min**	140	(96–203)	120	(79–161)	170	(114–227.75)	< 0.0001
**Cross clamp duration**	**Min**	74.5	(41–110)	66.5	(37–97)	79.5	(46–129.25)	0.003
**MV days**	**Days**	3	(2–6)	2	(2–4)	4	(2–8.5)	< 0.0001
**LOS**	**Days**	6	(4–13.5)	6	(4–14)	6	(4–13)	0.8
**Mortality**	**[n.]**	[11]		[1]		[10]		0.012
**Surgery**^**a**^	**[n.] 0**	[73]		[31]		[42]		0.003
	**1**	[306]		[157]		[149]		
	** > 1**	[41]		[9]		[33]		
**Cath lab**^**b**^	**[n.] 0**	[337]		[173]		[164]		0.53
	**1**	[73]		[20]		[53]		
	** > 1**	[10]		[4]		[7]		
**Medical**^**c**^	**[n.]**	[39]		[21]		[18]		0.61

*PIM3* Paediatric Index of Mortality3 score; *VIS* Vasoactive Inotropic Score, *basal SCr* basal serum creatinine, *CPB* cardiopulmonary bypass, *MV* mechanical ventilation, *LOS* length of stay

^a^Numbers of surgical procedures for each patient during the stay

^b^Numbers of cardiac catheterization laboratory procedures for each patient during the stay

^c^Medical admission: patients with no procedures during the stay

In case of missing BGA and vital signs data, a nonparametric missing value imputation algorithm was used: MissForest (MissForest R Package)—starting from the assumption of Missing at Random Case [13]. In case of laboratory missing data at the preset timepoint, the closest measure in time was selected. As far as fluids and therapies are concerned, in case of missing data, the input value was considered as zero. The only exceptions were patients with no arterial pressure data due to artifacts or errors in EHR system recording.

### Outcomes

#### Primary objective

To train and test an RF algorithm predicting the occurrence of severe AKI after 48 hours. This period has been established considering the possibility to reliably hypothesize a potential preventive intervention and considering that this is the time window within which KDIGO staging implies a creatinine modification. Furthermore, considering that KDIGO classification in our EHR refreshes hourly, but the most important changes occur every 6 hours (due to the urine output classification calculation), the predictive approach can be summarized using this schema:

- 6 h data [PREDICTION] > 48 h temporal delay > 6 h data [OUTCOME].

In other words, the information of all patients has been discretized in “6 h data” packages, and AKI has been predicted, continuously, in the time window of the first “6 h data” package, compared to the “6 h data” package occurring soon after the 48-h temporal delay and containing AKI classification information.

#### Secondary objectives

To train and test an RF algorithm predicting any AKI, mode AKI (the most common AKI stage of the six hourly assessments), and maximum AKI stage (the most severe AKI stage of the six hourly assessments) after 48 h; to detect the importance of each variable in the algorithm of AKI prediction (see “importance matrix” below); and to provide descriptive analyses of the sampled cohort.

### AI definitions

The RF algorithm proposed by Breiman [14] is an ensemble learning method for classification tasks that operates by constructing a multitude of decision trees at training time to reach a single result. Random forest is an improvement over bagged trees since it introduces a small tweak that decorrelates the trees [15]. The popularity of the RF method depends on its accuracy and on the fact that it can be applied to a wide range of prediction problems using a relatively small number of tuning parameters, compared to other methods [16].

The RF method graphs the predictors according to their effect on model improvement when splits are made on a predictor over the entire forest. The variable with the highest improvement score is set as the most important variable, and the other variables follow in order of importance (importance matrix). The variable importance scores are scaled to be between 0 and 100.

### Statistical analysis

#### Classification procedure and evaluation of the classification performance

The Classification And Regression Training (CARET) package of the R statistical software was used to create the predictive model through the classification procedure [17].

The classification procedure was set as follows:

- The dataset was split into train (70%) and test (30%) sets. The first was used to fit the classification model, whereas the latter was employed to evaluate its performance.
- All the reported results were obtained on the test dataset.
- In splitting the data, the percentages of each class were preserved in train and test sets.
- In order to have more stable predictions and avoid the problem of overfitting, the k–Fold Cross–Validation (with k = 10), repeated 3 times, was used.
- Tuning of the multiple try (mtry) parameter using the random choice option was performed to select the RF parameters.
- Finally, for this first model, the RF classification method was used.

The classification performance, according to the literature [18], was evaluated on different classification models:

- Binary classification with two possible classes.
- Multiclass classification with more than two classes.

Therefore, the KDIGO scores calculated in the EHR and targeted in the second 6-h window were discretized as follows:

- Bin AKI: 0 if AKI stages are zero, 1 otherwise.
- Severe AKI: 0 if the stages are all 0 or 1, 1 otherwise.
- Max AKI: the maximum value assumed within the 6 hourly measurements of the 6-h window.
- Mode AKI: the more frequent value within the 6 hourly measurements of the 6-h window.

The first two evaluations include binary values, while the other values are 0, 1, 2, and 3, following the KDIGO AKI stage criteria. Bin AKI and severe AKI are commonly present in the literature [19]. Max AKI and Mode AKI were introduced in the analysis to offer additional and complementary information to the previous ones. In the binary case, performance with the area under the curve receiver operating characteristic (AUC ROC) curve with 95% confidence interval (CI) was measured. Sensitivity, specificity, positive predictive value (PPV), and negative predictive value (NPV) were also analyzed. The multiclass mode performance was obtained through accuracy [20], computed through the confusion matrix and kappa index [21].

## Descriptive analyses

All continuous variables are expressed as median and interquartile range (IQR). Range was indicated in specific cases. When appropriate, mean and standard deviation were utilized. The Mann–Whitney test was used to assess the differences between no AKI/mild AKI and severe AKI populations. The chi-square test was utilized to compare the two groups. Kruskal–Wallis test and two-way analysis of variance were utilized to evaluate the modification of clinical variables over time.

A *p* value < 0.05 was considered statistically significant. Statistical analysis was performed with the GraphPad Prism 9.0 software package (GraphPad Software, San Diego, CA).

## Results

### Demographics of AKI incidence and outcomes

**Fig. 1:**
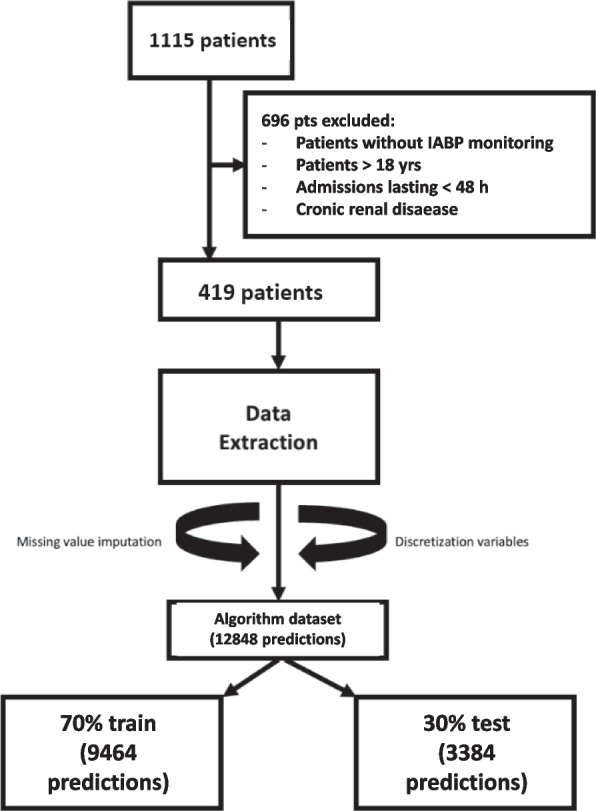
Patients’ enrollment and dataset building. IABP, invasive arterial blood pressure

During the study period, 1115 patients were admitted to the PCICU. Of these, 345 were excluded because of missing invasive arterial pressure data, and 351 were excluded because their admission lasted < 48 h. Therefore, 419 children were enrolled in the study. These patients were admitted to the PCICU after a cath lab procedure (*n* = 34), cardiac surgery (*n* = 299), cath lab and surgery (*n* = 48), and for medical reasons (*n* = 38) (Fig. 1). Demographic and clinical features are depicted in Table 2. Admission diagnoses and procedures are listed in supplementary Table 1.

### AKI incidence

Out of 419 patients, 71 (17%) had no AKI, 125 (30%) had stage 1, 75 (18%) had stage 2, and 148 (35%) had stage 3 AKI. Overall, 223 patients (53%) had a severe AKI episode. The diagnosis of AKI was made 1.4 (1–3) days after PCICU admission. AKI stage 1 was detected at a median of 1.7 (1.4–4.2) days from PCICU admission, AKI 2 at 1.4 (1–2.7) days, and AKI 3 at 1 (0.7–1.7) days. Using Kruskal–Wallis test, this difference was found to be statistically significant (*p* < 0.0001), with AKI stage 1 occurring later than other stages. Continuous renal replacement therapy was administered to eight patients (2%).

### PCICU length of stay and mortality

The average PCICU length of stay was 6 (4–14) days with a maximum stay of 275 days. The average duration of mechanical ventilation was 3 (2–6) days with a maximum of 93 days. A total of 11 (2.6%) patients died during PCICU admission.

### Characteristics of no/mild AKI and severe AKI patients

Severe AKI was only considered since it is the most consistent clinical categorization for epidemiological purposes in PCICU [19]. Several clinical variables showed significant differences between no/mild AKI patients and severe AKI patients (Table 2). Also, CPB and cross-clamping duration, basal serum creatinine, vasoactive inotrope score (VIS), and Pediatric Index of Mortality 3 (PIM3) showed significant differences between the examined populations (Table 2). The number of ventilation days and mortality were higher in severe AKI patients (Table 2). Fluid balances were frequently positive at postoperative day 1 (POD1) and tended to be negative thereafter (Supplementary Fig. 1). Using two-way ANOVA, there was a significant difference between fluid balances between no/mild AKI and severe AKI patients over time (*p* = 0.0003), with fluid balances being less negative in the latter group (Supplementary Fig. 1).

### Classification procedure and training performance

Along the 12,848 predictions, the RF model was developed on 70% of these, randomly selected. The test was applied to the remaining 30%. The train group and the test group did not show significant differences in overall demographic characteristics and model performance (data not shown).

Along 3854 predictions of the test population, bin AKI was classified with an AUROC of 0.93 (95% CI 0.92–0.94), a sensitivity of 0.71, specificity of 0.98, PPV of 0.92, and an NPV of 0.92. Severe AKI was classified by the RF model with an AUC ROC of 0.99 (95% CI 0.98–1), a sensitivity of 0.74, specificity of 0.99, PPV of 0.94, and an NPV of 0.97. Max AKI achieved 0.91 (k = 0.71), whereas mode AKI classification was 0.95 (k = 0.79). Confusion matrix of multiclass Max and Mode AKI are reported in Supplementary Fig. 2.

All variables used to train the algorithm are listed in Table 1.

Their importance in the algorithm efficiency was calculated through an importance matrix plot that provides a list of the most significant variables in descending order showing for each variable, how important it is in classifying the data.

### Importance matrix plot

The first five most important variables were:

- For binary AKI: creatinine, basal creatinine, platelets count, adrenaline support, and lactate dehydrogenase (LDH).
- For severe AKI: creatinine, CPB duration, basal creatinine, platelets, and LDH.
- For maximum AKI: creatinine, basal creatinine, platelets, LDH, and diuresis.
- For mode AKI: creatinine, basal creatinine, platelets, adrenaline, and LDH.

**Fig. 2:**
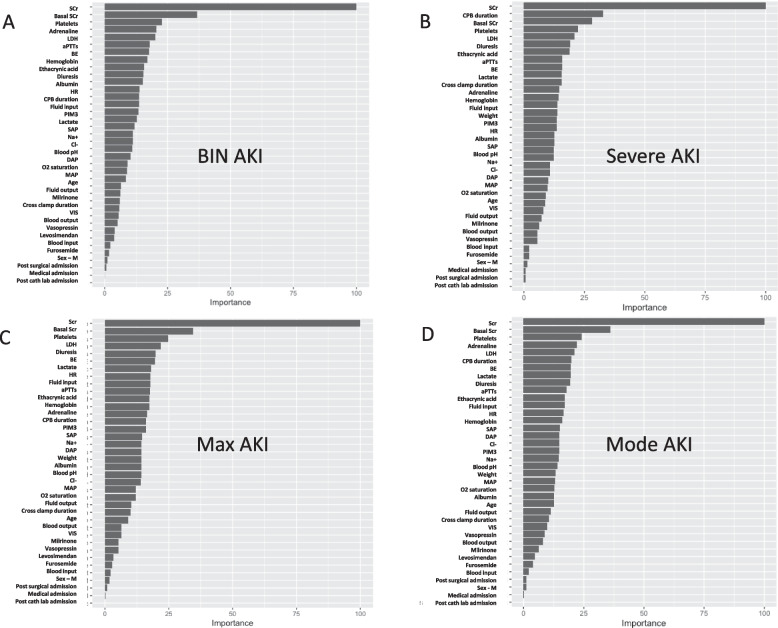
Importance matrix plots. **A** Binary acute kidney injury (AKI). **B** Severe AKI. **C** Max AKI. **D** Mode AKI

The remaining variables are shown in Fig. 2A–D.

## Discussion

The aim of the study was to implement a Machine Learning-driven AKI prediction algorithm in a PCICU setting and evaluate its performance on different severity of AKI.

AKI prevention is, so far, the action that can better improve morbidity and mortality caused by renal dysfunction, either in adults or pediatric critical patients. Therefore, relying on ML algorithm to detect patients who can benefit from a kidney protective strategy can be successful in decreasing AKI rates. Considering the high performance of these methods in predicting a potentially avoidable complication, this is potentially a powerful tool to improve clinician workflow, ultimately leading to more personalized and efficient health care.

This study confirms that AI can be a feasible and accurate tool for the continuous prediction of AKI in pediatric cardiac patients; in binary and in multiclass classification, its performance is good. Furthermore, the RF algorithm is well recognized for its accuracy and its ability to deal with both small sample sizes and high-dimensional feature spaces.

As highlighted by Biau and Scornet [16], the popularity of RF depends on the fact that they can be applied to a wide range of prediction issues using a relatively small number of tuning parameters, compared to other methods. The presence of a little number of parameters to tune makes using this algorithm easier than others.

The variable importance plot gives an indication of how useful the variables are for prediction.

Interestingly, in importance matrix plots shown for every classification, distribution variables were rather consistent, with the surgical status (both post-surgical, post-cath lab, and medical) being the least important and creatinine, platelets, and LDH and diuresis being among the most influential. While the importance of creatinine and diuresis is not surprising, it is interesting that platelets and LDH are constantly on top of the plot, while other variables are relevant in some classifications (CPB duration in severe AKI, adrenaline in binary AKI), but not in others. It can be speculated that LDH is derived from hepatocytes and liver congestion as a response to right ventricular dysfunction, which is frequently associated with AKI [22]. Also, LDH is an important marker of systemic (cellular) perfusion and possible indicator of hemolysis [23]. In one case, it implies AKI due to impaired anterograde perfusion; on the other hand, renal toxicity is a sequela of potential free hemoglobin [24]. However, it appears as a novel biomarker, and this could be one of the results of AI applications. Similarly, the other included parameters, whose importance may appear as scaled down (i.e., fluid input and fluid output) or that appeared significant at “traditional” univariate analysis, should be seen in the context of an ML method, where all the predictors must be integrated in the calculation. Finally, once again, the application of serial creatinine measurements (including basal values, always available in cardiac patients), although long-known as a biased biomarker, is confirmed as fundamental in predicting AKI in this subset [25]. It might be interesting to appraise if the integration of a routinely available renal biomarker might further improve the model.

This model was particularly efficient in detecting severe AKI (meant as stages 2 and 3) that is the most common outcome of observational studies [4]. The multiclass prediction of max AKI and mode AKI is little explored in the literature but may offer complementary information to the binary one to support physicians’ decisions. As a matter of fact, not only can the occurrence of AKI be predicted, but also its severity or its most frequent occurrence along the KDIGO stages, and this could be determinant since specific bundles for mild or severe AKI could be designed.

ML models, e-alert systems, and clinical decision support systems have been variably described in the pediatric literature with controversial results [26]. In general, they have been shown to reduce the time to AKI diagnosis in several clinical settings, including in patients with cardiac disorders. However, these methods frequently resulted in false positives, leading to unnecessary interventions or unintended harm [27]. Evidence showing improved outcomes with ML prediction models for pediatric AKI is currently scarce. Moreover, ML models may be difficult to understand by bedside clinicians. Furthermore, significant data cleaning must be performed to develop these algorithms, and management of missing data can affect the calculations. Urine output is also frequently excluded from ML models due to difficult data retrieval [26].

In this light, our system would have significant positive characteristics. It applied the automatic AKI diagnosis calculation, available in our center, that accurately and timely identified all AKI stages, including urine output measures. Furthermore, data cleaning was relatively unnecessary, and the amount of missing data was limited by choosing predictors that were all widely available in our EHR. Finally, false positives in our system could be reduced by avoiding the prediction of mild AKI or infrequent episodes (i.e., by choosing Mode AKI and not Max or severe AKI as an outcome to predict).

This AI model has the potential to be applied to the EHR as an advanced e-alert, which automatically and continuously provides the AKI risk in all patients, according to each of the classification methods. The future direction will be to implement this system to verify if clinicians would act differently by knowing that an AKI risk is relevant in their patients. A potential alternative might be to create a dedicated bundle of actions aiming at AKI prevention.

### Limitations

Major limitations of the study are its retrospective nature, the small sample size, and the lack, among the variables, of some recognized risk factors involved in the kidney injury process after cardiac surgery (i.e., vancomycin administration, aminoglycosides prescription, surgical risk score, cardiac anatomy, and emergency admission). We did not include such risk factors because they were frequently lacking in our EHR database. On the 36 variables extracted, there was almost no need for data cleaning, and missing variables were reliably managed. Finally, this study requires an external and prospective validation to consistently confirm these preliminary data. A similar set of children in another PCICU or a novel set of patients admitted after the studied period should be included and enrolled in a novel study. This work mainly aimed to verify the feasibility of EHR consultation and data selection, download, and managing. Validation of the model is going to be soon applied, considering the encouraging results of the training and test.

Lastly, we did not consider if the model could distinguish transient AKI cases and persistent AKI cases (i.e., lasting less or more than 48 h). However, in a recent observation (4), our group detected a relatively low rate of persistent AKI in pediatric cardiac surgery patients (3.5%) and it is possible that in a future larger study this outcome may be detected.

## Conclusions

An ML model to detect severe AKI occurring after 48 h in PCICU patients showed with high accuracy that creatinine, basal creatinine, platelets count, adrenaline support, and LDH are the most important predictors. Any AKI, the most frequent level of AKI (mode AKI), and the maximum level can also be predicted. This algorithm can help clinicians in individuating patients at risk of AKI in which a preventive strategy can be attempted.

### Supplementary Information

**Additional file 1: Supplementary Table 1.** Stratification of population by diagnoses and procedures.

**Additional file 2: Supplementary Figure 1.** Fluid balances in acute kidney injury (AKI) and no AKI patients in the first seven postoperative (POD) days.

**Additional file 3: Supplementary Figure 2.** Confusion matrixes. Panel A) Max acute kidney injury (AKI); Panel B) Mode AKI.

## Data Availability

The dataset used and analyzed during the current study is available from the corresponding author upon reasonable request.
