# Supplementary material for: Predicting acute kidney injury with an artificial intelligence-driven model in a pediatric cardiac intensive care unit
Source: J Anesth Analg Crit Care. 2023 Oct 18;3:37. doi: 10.1186/s44158-023-00125-3 (PMC10583404; doi:10.1186/s44158-023-00125-3)
Supplement: Supplementary file 1 — Additional file 1: Supplementary Table 1. Stratification of population by diagnoses and procedures. [file 44158_2023_125_MOESM1_ESM.docx]

**Supplementary table 1. Stratification of population by diagnoses and procedures.**

| **DIAGNOSES** | **N.** | **PROCEDURES** | **N.** |
| --- | --- | --- | --- |
| Airways Anomaly (any type) | 8 | Airways Surgery | 6 |
| Aortic Valve Regurgitation | 2 | Corrective surgery (all kind of biventricular repairs) | 188 |
| Aortic Valve Stenosis | 16 | Diagnostic Cath Lab Procedure | 10 |
| ASD | 7 | ECMO/VAD | 13 |
| ASD+VSD | 1 | Hybrid Palliation | 4 |
| AVB/Arrhythmias | 14 | Interventional Cath Lab Procedure | 27 |
| CAVC | 11 | No surgical/cath lab procedures | 36 |
| CCTGA | 6 | Norwood Operation | 5 |
| CMP | 23 | PAB | 13 |
| CMP (admitted for VAD/Tx) | 11 | Pacemaker | 11 |
| Coarctation of Aorta | 15 | Redo (any redo operation on biventricular CHDs) | 42 |
| Coarctation+VSD | 17 | Shunt | 14 |
| Coronary Anomaly | 3 | Stage II-III Palliation^a^ | 17 |
| DORV | 4 | Transplant (Heart, Lung, Heart-Lung) | 11 |
| EBSTEIN Disease | 2 | Unifocalization | 1 |
| HLHS (includes Shone complex, then palliated) | 29 | Other* | 21 |
| LUNG (primary lung disease, includes PH) | 5 | **Total** | **419** |
| Mitral Valve Regurgitation | 6 |  |  |
| Mitral Valve Stenosis | 5 |  |  |
| Neoplasia (all intrathoracic sites) | 3 |  |  |
| PA+VSD+MAPCAs | 15 |  |  |
| Partial AVSD | 5 |  |  |
| PDA | 2 |  |  |
| Pericardial effusion | 1 |  |  |
| Pulmonary Valve Stenosis | 13 |  |  |
| PAPVR | 2 |  |  |
| TAPVR | 1 |  |  |
| Severe Coarctation (includes IAA) | 9 |  |  |
| TGA | 38 |  |  |
| TOF | 53 |  |  |
| Truncus Arteriosus | 10 |  |  |
| UVH (all univentricular anatomies except for HLHS) | 23 |  |  |
| Vascular ring | 6 |  |  |
| VSD | 43 |  |  |
| Other* | 10 |  |  |
| **Total** | **419** |  |  |

“Procedure” is intended as the first one to whom each patient underwent during the stay. Each patient’s data were collected from the beginning of the ICU stay (no matter if postoperative/post cath lab/medical) until the discharge or any other second procedure.

*any diagnosis or procedure that could not be classified in the above mentioned.

ASD= Atrial Septal Defect; ASD+VSD= Atrial Septal Defect + Ventricular Septal Defect; AVB= Atrioventricular Block; CAVC= Complete Atrioventricular Canal defect; CCTGA= Congenitally Corrected Transposition of the Great Arteries; CMP= Cardiomyopathy, including dilative, hypertrophic, obstructive; CMP (admitted for VAD/Tx)= all End Stage Heart Failure admitted for any Ventricular Assist Device or Transplant; DORV= Double Outlet Right Ventricle; HLHS= Hypoplastic Left Heart Syndrome; PH= Pulmonary Hypertension; PA+VSD+MAPCs= Pulmonary Atresia + Ventricular Septal Defect + Major Aortopulmonary Collateral Arteries; PAVSD= Partial Atrioventricular Septal Defect; PDA= Patent Ductus Arteriosus; PAPVR= Partial Anomalous Pulmonary Venous Return; TAPVR= Total Anomalous Pulmonary Venous Return; IAA= Interrupted Aortic Arch; TGA= Transposition of the Great Arteries; TOF= Tetralogy Of Fallot; UVH= Univentricular Heart; VSD= Ventricular Septal Defect; ECMO/VAD= Extracorporeal Membrane Oxygenation/Ventricular Assist Device; PAB= Pulmonary Artery Binding; CHD= Congenital Heart Disease; a=Glenn and Fontan procedures.
