# Supplementary figures and images for: Predicting acute kidney injury with an artificial intelligence-driven model in a pediatric cardiac intensive care unit
Source: J Anesth Analg Crit Care. 2023 Oct 18;3:37. doi: 10.1186/s44158-023-00125-3 (PMC10583404; doi:10.1186/s44158-023-00125-3)

## Slide 1
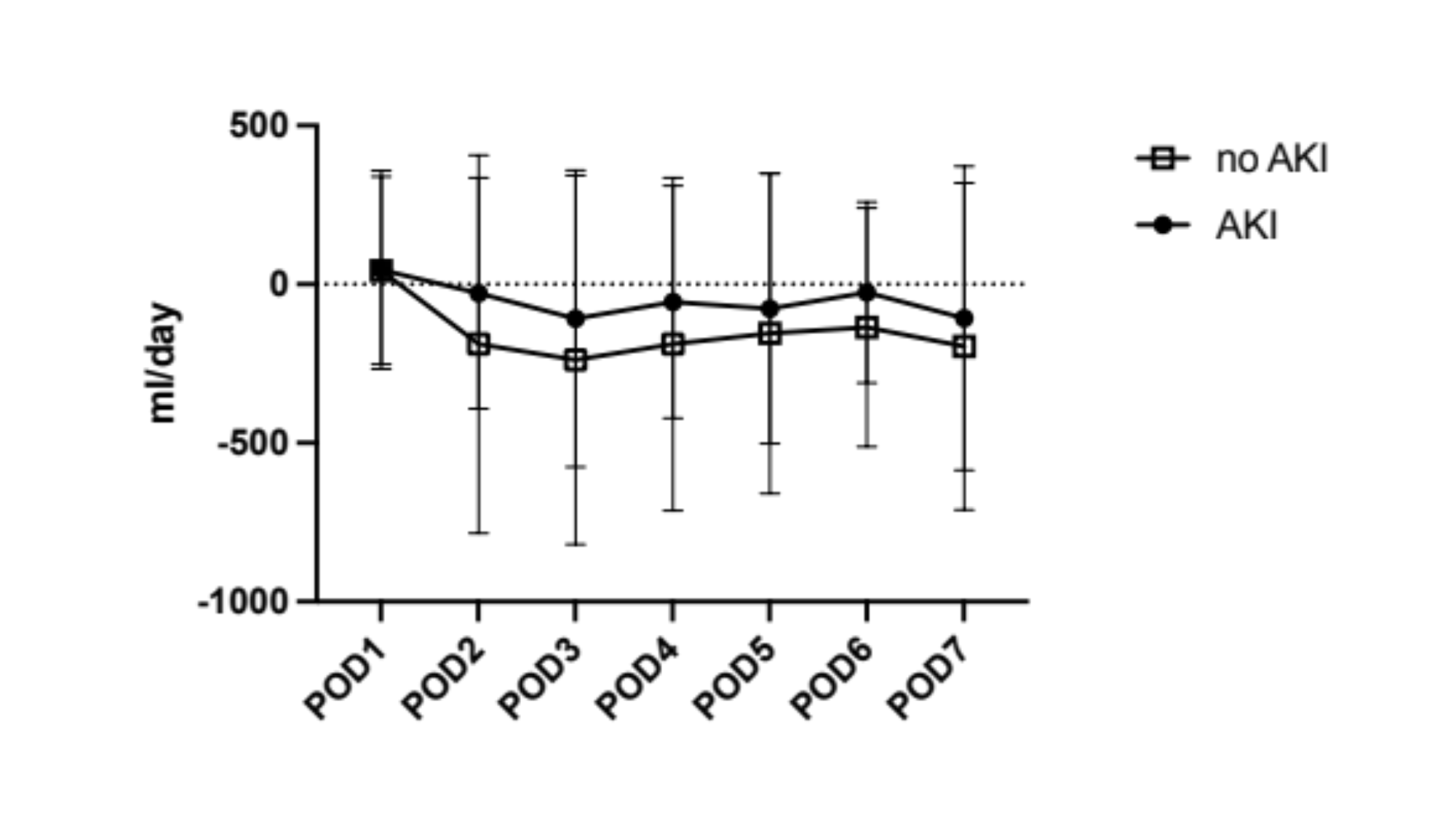

Supplement: Supplementary file 2 — Additional file 2: Supplementary Figure 1. Fluid balances in acute kidney injury (AKI) and no AKI patients in the first seven postoperative (POD) days. [file 44158_2023_125_MOESM2_ESM.pptx]

## Slide 1
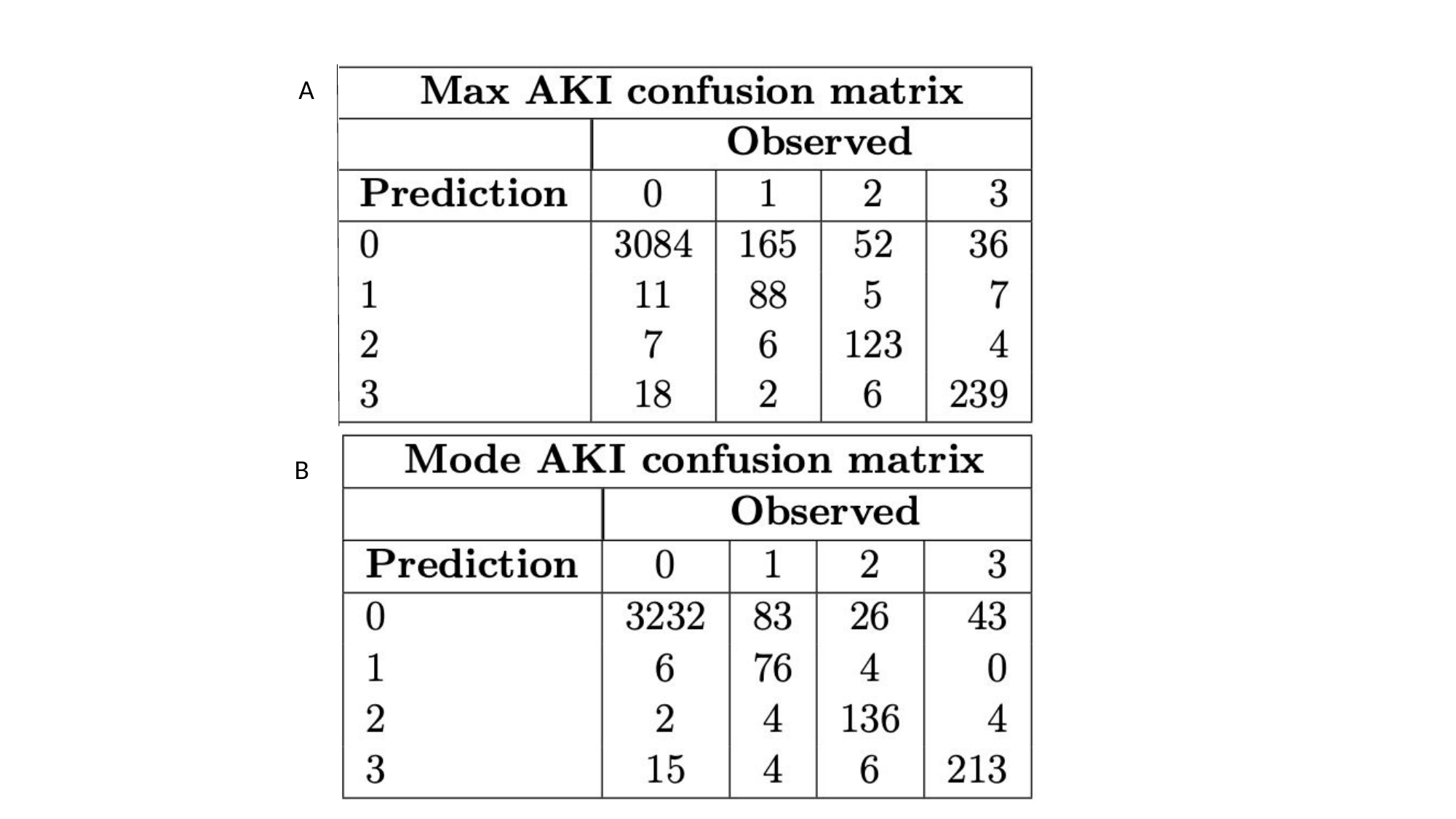

A
B

Supplement: Supplementary file 3 — Additional file 3: Supplementary Figure 2. Confusion matrixes. Panel A) Max acute kidney injury (AKI); Panel B) Mode AKI. [file 44158_2023_125_MOESM3_ESM.pptx]
